# Economic considerations support C-reactive protein testing alongside malaria rapid diagnostic tests to guide antimicrobial therapy for patients with febrile illness in settings with low malaria endemicity

**DOI:** 10.1186/s12936-019-3059-5

**Published:** 2019-12-26

**Authors:** Yoel Lubell, Arjun Chandna, Frank Smithuis, Lisa White, Heiman F. L. Wertheim, Maël Redard-Jacot, Zachary Katz, Arjen Dondorp, Nicholas Day, Nicholas White, Sabine Dittrich

**Affiliations:** 1 Mahidol-Oxford Tropical Medicine Research Unit (MORU), Faculty of Tropical Medicine, Mahidol University, Bangkok, Thailand; 2 Nuffield Department of Clinical Medicine, Centre for Tropical Medicine and Global Health, University of Oxford, Oxford, UK; 3 Department of Clinical Research, Faculty of Infectious and Tropical Diseases, London School of Hygiene & Tropical Medicine, London, UK; 4 Myanmar Oxford Clinical Research Unit, Yangon, Myanmar; 5 Department of Medical Microbiology, Medical Center for Infectious Diseases, Radboud University, Radboudumc, Nijmegen, The Netherlands; 6 Foundation of Innovative New Diagnostics (FIND), Geneva, Switzerland

**Keywords:** C-reactive protein, Malaria, Rapid diagnostic test, Point-of-care test, Febrile illness, Cost-effectiveness

## Abstract

Malaria is no longer a common cause of febrile illness in many regions of the tropics. In part, this success is a result of improved access to accurate diagnosis and effective anti-malarial treatment, including in many hard-to-reach rural areas. However, in these settings, management of other causes of febrile illness remains challenging. Health systems are often weak and other than malaria rapid tests no other diagnostics are available. With millions of deaths occurring annually due to treatable bacterial infections and the ever increasing spread of antimicrobial resistance, improvement in the management of febrile illness is a global public health priority. Whilst numerous promising point-of-care diagnostics are in the pipeline, substantial progress can be made in the interim with existing tools: C-reactive protein (CRP) is a highly sensitive and moderately specific biomarker of bacterial infection and has been in clinical use for these purposes for decades, with dozens of low-cost devices commercially available. This paper takes a health-economics approach to consider the possible advantages of CRP point-of-care tests alongside rapid diagnostic tests for malaria, potentially in a single multiplex device, to guide antimicrobial therapy for patients with febrile illness. Three rudimentary assessments of the costs and benefits of this approach all indicate that this is likely to be cost-effective when considering the incremental costs of the CRP tests as compared with either (i) the improved health outcomes for patients with bacterial illnesses; (ii) the costs of antimicrobial resistance averted; or (iii) the economic benefits of better management of remaining malaria cases and shorter malaria elimination campaigns in areas of low transmission. While CRP-guided antibiotic therapy alone cannot resolve all challenges associated with management of febrile illness in remote tropical settings, in the short-term a multiplexed CRP and malaria RDT could be highly cost-effective and utilize the well-established funding and distribution systems already in place for malaria RDTs. These findings should spark further interest amongst industry, academics and policy-makers in the development and deployment of such diagnostics, and discussion on their geographically appropriate use.

## Background

### Challenges and opportunities in the management of patients testing negative for malaria

The global malaria burden has halved since the turn of the century, mainly due to improved access to diagnosis, treatment and insecticide-treated nets. Progress, however, has stalled, with no reduction in cases between 2015 and 2017 [1]. In 2017, funding for malaria reached a new high, exceeding US$3 billion, and worldwide there is appetite to eliminate malaria [2, 3]. Consequently, substantial economic opportunity exists for interventions that can capitalize on the global commitment to eradicate malaria, although a protracted era of a low but persistent burden could see this momentum lost. Coupled with the insidious spread of artemisinin resistance, this could have dire consequences in the form of resurgent drug resistant malaria.

**Fig. 1:**
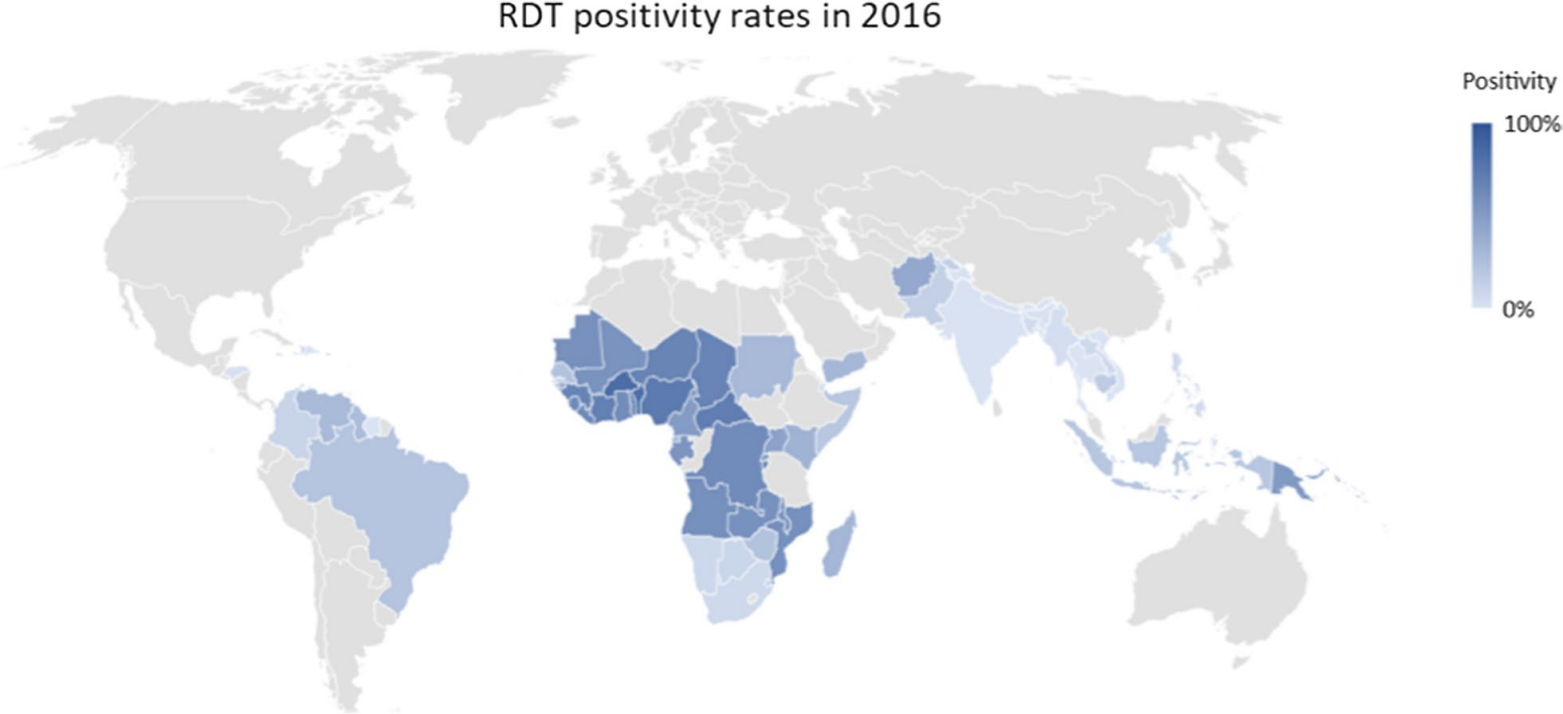
The percentage of malaria rapid diagnostic tests with a positive result [5]

Nearly 250 million malaria rapid diagnostic tests (RDTs) were distributed in 2017, almost quadruple the number distributed in 2010 [1]. In Asia, Latin America and increasingly in Africa, most patients tested with an RDT have a negative result (Fig. 1) [4]. In the Asia-Pacific, only 3% of over 25 million RDTs were positive for malaria in 2016. For the vast majority of patients in whom malaria has been ruled out, no other diagnostic tools are available, and clinical algorithms for their management have shown moderate and inconsistent performance [5, 6], although improvement has been shown to occur with their adaptation to an electronic format [7, 8]. Utilization of the malaria RDT infrastructure—both the devices themselves, as well as their funding and distribution networks, could offer an efficient and scalable pathway to improved management of febrile illness.

There are increasingly urgent reasons for such a strategy [9]. First, are the potentially adverse health outcomes for the increasingly large proportion of patients testing negative for malaria: fever studies in Southeast Asia demonstrate that up to a third of febrile patients with a bacterial infection go unrecognized [10, 11], some with pathogens associated with high mortality when untreated [12, 13]. Second, is the increasing proportion of patients with a viral infection receiving antibiotics, promoting spread of antimicrobial resistance (AMR) [11, 14–16]. Finally, as malaria declines patients have little incentive to seek care from health workers that cannot test or treat other diseases; integrated management of febrile illnesses has been shown to increase patient attendance at formal health providers, contributing to improved malaria case detection and elimination efforts [17].

### A potential solution: host biomarker-guided antibiotic therapy in febrile illness

One strategy with the potential to address all three of these issues is the use of host biomarker testing to identify patients most likely to benefit from antibiotic treatment. Use of such tests in conjunction with up-to-date aetiological surveillance data could further enhance the impact and cost-effectiveness of this approach [18]. An ideal biomarker of bacterial infection would be robust to temporal and spatial heterogeneity in causes of fever [11], and will, therefore, have been studied in a broad range of populations.

Numerous biomarkers to identify bacterial infections have been proposed [19], but none have perfect sensitivity and specificity. The most studied is C-reactive protein (CRP), a marker of inflammation that has been in clinical use for decades [20]. While in some populations it has underperformed, particularly at the severe end of the clinical spectrum [21], in many studies CRP has been found to be appropriately sensitive and moderately specific to identify patients with a bacterial infection [22, 23]. Low-cost and accurate rapid CRP tests appropriate for use in remote tropical settings are widely available [24–26].

A 2014 Cochrane review confirmed the utility of CRP-guided antibiotic therapy in patients with non-severe acute respiratory infections (ARIs) [27]. This approach is routine in Scandinavian countries and the Netherlands, and recommended in guidelines from the National Institute for Clinical Excellence (NICE) in England and Wales [28]. A recent clinical trial in Vietnam demonstrated a 20% point reduction in antibiotic prescription in primary care patients with non-severe ARIs [29], and a secondary analysis of a clinical trial in Tanzanian children with non-severe ARIs demonstrated that when deployed together with a clinical algorithm, CRP-guided treatment can reduce antibiotic prescriptions and improve clinical outcomes [30]. Fewer studies investigate the utility of CRP-guided antibiotic therapy in the general febrile population. A retrospective analysis of 1300 febrile patients across Southeast Asia found CRP to be a sensitive marker of bacterial infection [31]. A clinical trial of CRP-guided treatment in patients with non-severe febrile illnesses in primary care in Thailand and Myanmar demonstrated reductions in antibiotic prescribing [32].

This paper provides preliminary, ‘broad brush’ economic analyses of the impact of CRP tests to guide antibiotic prescription in patients with febrile illness, considering (i) direct benefit to individual patients with bacterial illnesses; (ii) societal benefits in terms of the costs of AMR averted; and (iii) the impact on malaria case detection and elimination efforts. A combined rapid test for malaria and CRP could harness existing infrastructure and funding already in place to support distribution of malaria RDTs, with one such test commercially-available and undergoing analytical field validation [33]. Access related factors favouring combined malaria-CRP tests are also explored, namely synergistic procurement pathways, and the large market share which could make such tests attractive to developers, ensuring long-term sustainability, independent of international donor funding.

It is important to emphasize that the analyses are kept deliberately simple for non-economist readers and the data to support them are often scarce and drawn from a variety of settings. Further adaptation and contextualized analyses will be required if such multiplex devices are to be considered as replacements for malaria RDTs in different geographical settings.

### Direct health benefits for patients with bacterial illnesses

**Fig. 2:**
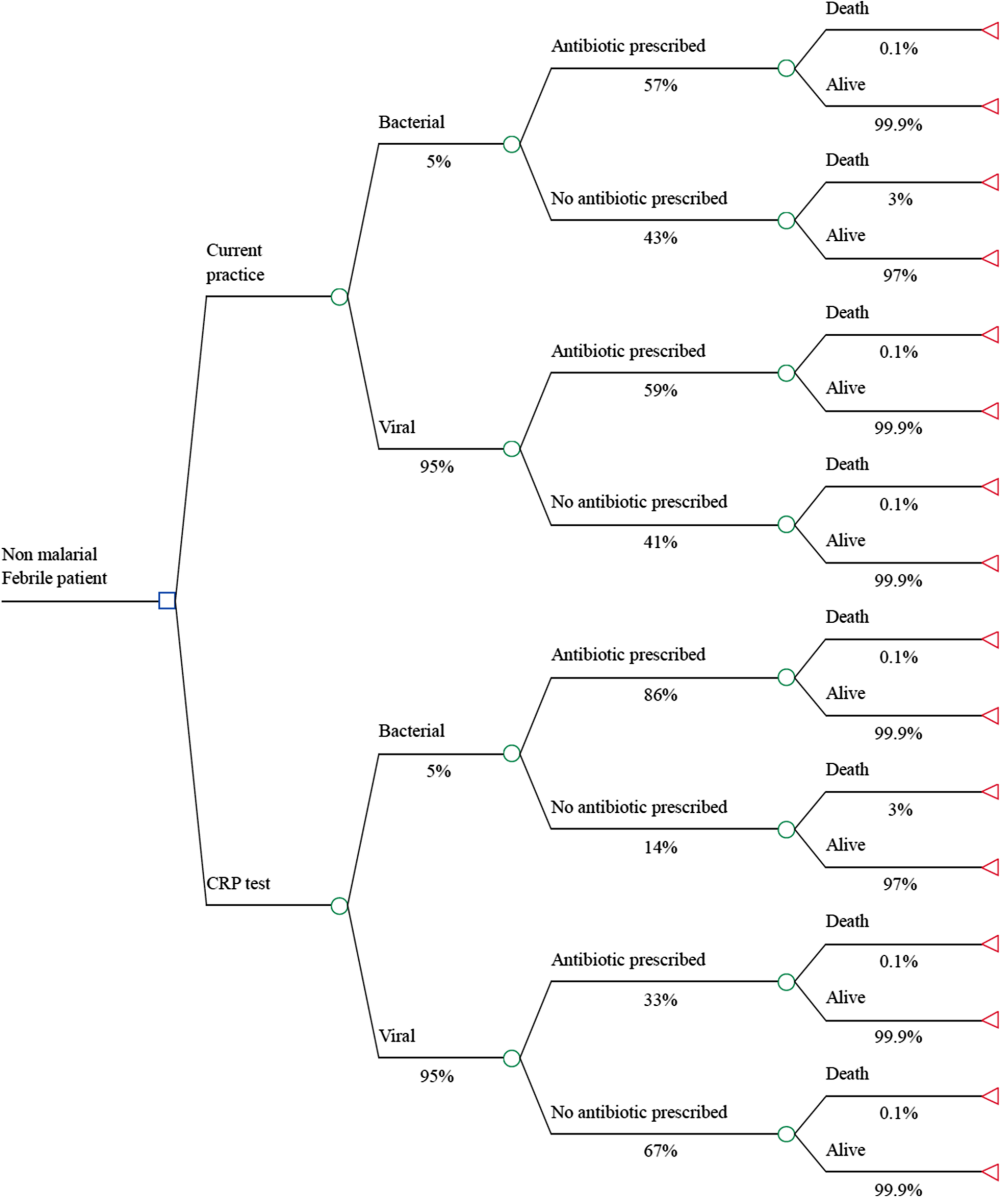
Decision tree for management of febrile patients with a negative malaria RDT result

To consider the potential impact of better identification and treatment of bacterial infections data from the World Malaria Report is drawn on to estimate the number of RDTs negative for malaria [4]. A simple decision tree (Fig. 2) is used to incorporate the proportion likely due to bacterial infection, estimated at 5% [15, 34, 35]; and the probability these are diagnosed and treated based on clinical judgement, set at 57% [11]. The sensitivity of a CRP test with a threshold of 20 mg/l to detect bacterial infections, is estimated at 86% [31]. For untreated bacterial infections a conservative mortality rate of 3% (half the median estimate from a Delphi survey on this topic) is assumed [36]. The added cost of a combined malaria-CRP test is estimated at $1 on top of that of a malaria RDT, and this additional cost is applied to all patients, irrespective of the malaria test result.

With these assumptions applied to the 25 million febrile episodes in which malaria RDTs were used in the Asia-Pacific in 2016, CRP-guided antibiotic treatment in the 97% of malaria negative cases could avert approximately 10,500 deaths per year due to better identification of patients with bacterial infections. Assuming an additional cost of $1 per combination test, the incremental cost effectiveness ratio (ICER) per disability adjusted life year (DALY) averted would be $28, therefore considered a highly cost-effective intervention.

### Costs of antimicrobial resistance averted

The same decision tree structure is used to consider reductions in unnecessary antibiotic prescribing, averting both direct antibiotic costs and, more importantly, indirect health and economic costs of emergence and spread of AMR. A recent analysis provided rudimentary estimates for the cost of AMR per antibiotic consumed [37]. In the Thai context the consumption of a course of broad spectrum beta-lactam antibiotics was associated with a cost of AMR of approximately $10. In this analysis, this is adjusted downwards to $3.8 using the ratio of GDP per capita (PPP) for all endemic countries in the Asia-Pacific compared with that of Thailand, weighted by the volume of RDTs used in each country [38]. The costs of AMR averted are then estimated if all patients with low CRP test results (a CRP level of less than 20 mg/L) were not prescribed an antibiotic. These estimates for the costs of AMR averted are then applied alongside direct purchase costs of the antibiotics and the incremental costs of CRP tests, to calculate the net-benefit of CRP-guided antibiotic therapy.

Under current practice, amongst the 24.5 million patients with a negative malaria RDT, 59% would be prescribed an antibiotic (corresponding closely with a recent review on the topic [39]). With CRP guidance, antibiotic prescribing in patients with a negative malaria test would reduce to 36%, averting approximately 5.6 million courses of antibiotics annually in the Asia-Pacific. Considering the costs of CRP tests and the direct and indirect costs of antibiotics, combined malaria-CRP tests would be expected to be cost-saving, with a net-benefit of over USD $9.5 million.

### Higher malaria detection and treatment to support malaria elimination

**Fig. 3:**
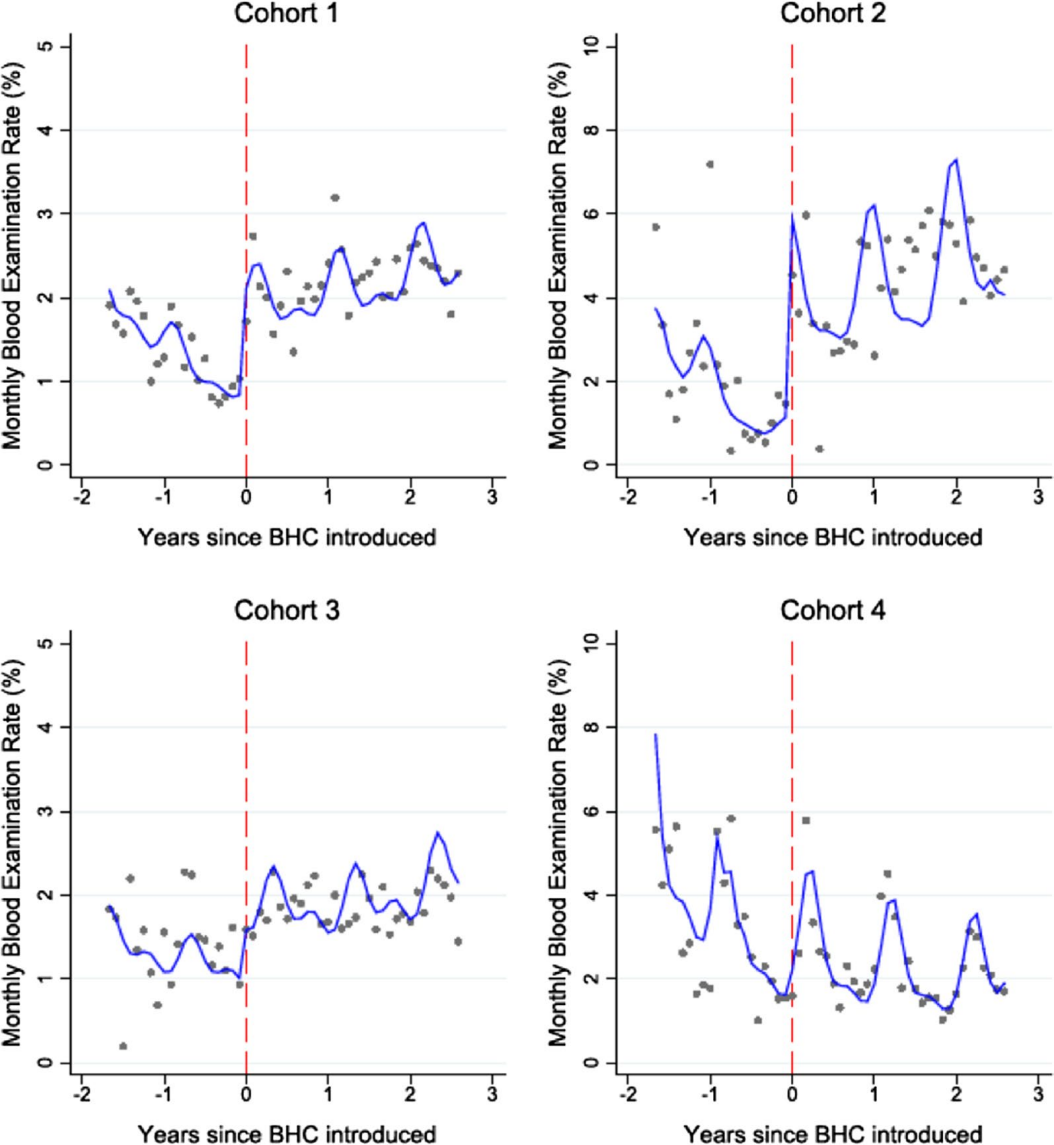
Monthly blood examination rates in four cohorts of Community Health Workers in 154 villages in rural Myanmar before and after the introduction of other basic health services. Grey dots represent observed aggregated data and blue lines indicate the predictions from a mixed effects negative binominal regression model. The vertical red line denotes the time when the basic health care package was introduced [17]

Finally, the benefit of improved malaria case detection in areas targeting elimination is considered. This was demonstrated in the context of community health workers (CHWs) in Myanmar, initially introduced only to diagnose and treat malaria. Over time, malaria testing rates steadily declined as febrile patients benefitted little from having malaria excluded without other treatment for their illness. Medical Action Myanmar, an organization supporting a network of CHWs, extended the role of the CHWs to include a broader range of basic health services [17]. This was followed by an immediate and sustained increase in testing rates (Fig. 3).

The health benefits for patients with bacterial illnesses were described above, but in addition to these are the direct health benefits for patients with malaria, due to the higher testing (and treatment) rates. A simple cost-effectiveness calculation for the potential benefits of better malaria case detection, by virtue of increasing the probability that a febrile patient will present to a health worker, demonstrates that malaria-CRP rapid tests would be cost-effective ($36 per DALY averted), even when considering only the improved outcomes for the small proportion of patients with malaria (Table 1).

**Table 1:** Cost per DALY averted considering higher malaria testing and treatment rates

	Introduction of treatment for NMFIs	Before	After	Sources/estimation/comments
A	Median village population	768		McLean [17]
B	Monthly malaria tests carried out	15.0	34.9	McLean [17]
C	% testing positive for malaria	9.2%	6.0%	McLean [17]
D	Monthly incidence NMFI (regional average)	38.4		Capeding [42]
E	Monthly incidence malaria	3.9	2.4	(D * C)/(1 − C)
F	Total incidence febrile illness	42.3	40.8	D + E
G	Probability febrile patient attends CHW	35%	86%	B/F
H	Treated malaria cases	1.4	2.1	B * C
I	Untreated malaria cases	2.5	0.4	E − F
J	Untreated malaria cases averted		2.2	∆I
K	Mortality rate in untreated malaria	1%		Lubell [36]
L	Monthly malaria mortality averted per village		0.029	J * K
M	Years of life lost per death	45		Assumption
N	DALYs averted		0.97	L * M
O	Cost per CRP test	$1		CRP test costs are as low as $0.5; additional costs of transport, storage and training included here
P	Incremental cost per month		$ 35	0*B2
	*ICER (cost per DALY averted)*		*$ 36*	*P/N*

**Fig. 4:**
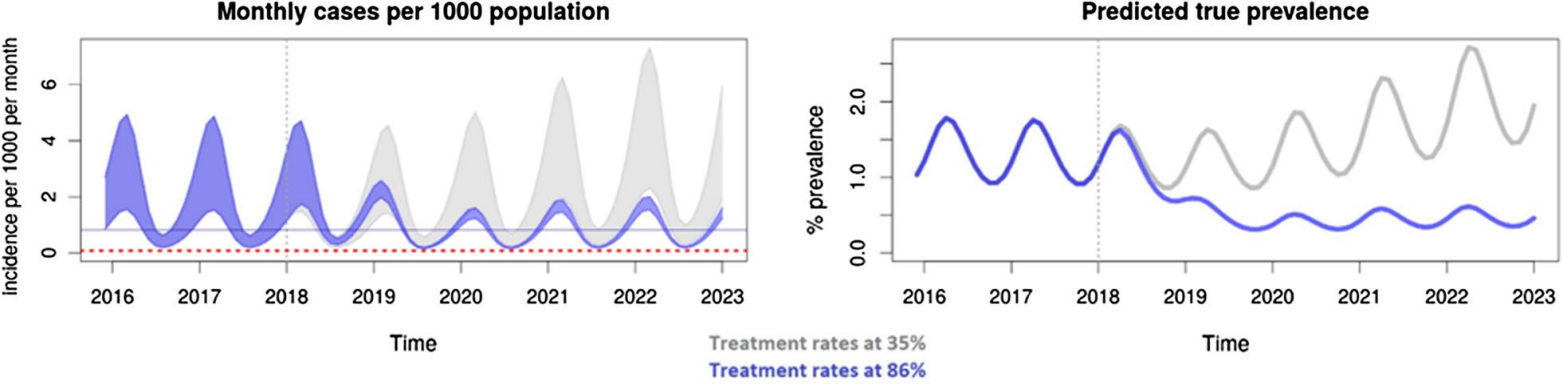
Predicted prevalence and incidence of malaria in two scenarios with the only difference being the proportion of cases that are detected and treated, varying these from 35 to 86%. The grey solid line illustrates the baseline scenario and the blue solid line is the elimination strategy scenario. The dark blue solid line is the target baseline Annual Parasite Incidence (API). The grey dashed line indicates the start of elimination activities. The red dashed line is the pre-elimination threshold (an API of 1 per 1000 per year)

In addition to direct benefits to patients with malaria are the implications of higher case detection rates on time to interruption of transmission. This is illustrated by a previously described model [40] for which a web-based interface is available for further simulation. Increasing malaria case detection rates is predicted to reduce subsequent malaria incidence and prevalence, implying faster time to interruption of transmission, resulting in shorter malaria elimination campaigns and further cost-savings (Fig. 4).

### Market incentive for global investment

The above health and economic benefits could be achieved with commercially available rapid CRP tests. However, while this is a workable approach, long-term success is more likely if existing malaria control infrastructure (for example, training, procurement channels and supply chains) are leveraged using a combined malaria-CRP test; the anticipated market opportunity for such a device is explored below.

**Fig. 5:**
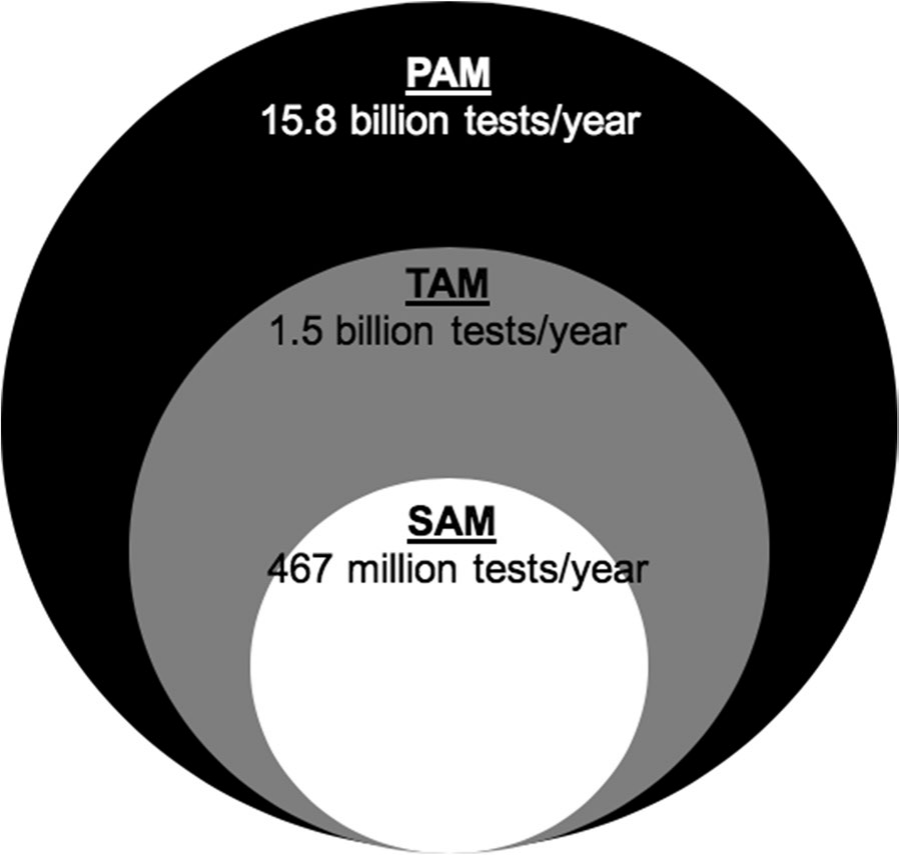
Market analysis to estimate the market size of a malaria-CRP combination test. *PAM* potential available market, *TAM* total available market, *SOM* serviceable and obtainable market

Unitaid’s annual global forecast estimates that 15.8 billion febrile episodes occur amongst individuals living in malaria endemic low- and middle-income countries (LMICs) [41]. This indicates a large potential available market (PAM) for a malaria-CRP test. From the PAM, the total available market (TAM) can be calculated, which accounts for the number of febrile patients likely to seek care. Treatment seeking rates from household surveys indicate an annual TAM of 1.48 billion tests. Within the TAM, the currently accessible market, termed the Serviceable Available Market (SAM), is determined by the yearly number of malaria RDTs and microscopy slides performed within LMICs where malaria is prevalent [4]. In 2016, the SAM comprised approximately 467 million tests (Fig. 5).

Finally, to produce a pragmatic demand forecast reflective of probable market consumption, the serviceable and obtainable market (SOM) must be estimated, by identifying and analysing factors that might affect in-country adoption, including malaria prevalence, existing market size for substitutes, planned procurement mechanisms, government commitment to universal health care, malaria financing and price of diagnostics. These factors must be explored in detailed discussions with procurement and government agencies. A simple SOM estimate can be calculated by considering two key factors; countries with a low proportion of malaria infections amongst the febrile population (less than 10%); and those with strong existing malaria RDT markets, as opposed to reliance on blood film microscopy. Although incorporating additional factors would reduce the SOM, considering these two factors identifies more than 23 LMICs (mainly in Southeast Asia and sub-Saharan Africa), in which more than 55 million malaria RDTs were sold in 2016.

## Conclusion

While multiple approaches exist to improving care for patients with febrile illness, the strategy described in this paper could be rolled out in the near-term using existing infrastructure and capitalizing on current global appetite for malaria elimination. The rudimentary analyses presented here provide a general indication that integrating CRP-guided antibiotic therapy with malaria RDTs is likely to be highly cost-effective considering either (and of course all) the potential benefits in the different analyses, although further refinement and context-specific adaptations to these analyses are required to verify their conclusions. While CRP-guided antibiotic therapy is not without limitations it could offer considerable advantages over current practice, and pave the way for rapid implementation of improved technologies as they become available. In the longer term better biomarkers and multiplexed pathogen-specific tests, deployed with the support of electronic clinical decision algorithms, could replace CRP-guided therapy, but the timeline for this is likely to be long, with substantial preventable mortality, morbidity and erosion of antibiotic resources in the interim. To avoid this, low-cost CRP tests could be deployed alongside malaria RDTs or incorporated into a combination point-of-care test.

## Data Availability

The datasets used and/or analysed during the current study are available from the corresponding author on reasonable request.

## Abbreviations

AMR: antimicrobial resistance
ARI: acute respiratory infection
CHW: community health worker
CRP: C-reactive protein
DALY: disability adjusted life year
GDP: gross domestic product
ICER: incremental cost effectiveness ratio
LMIC: low- and middle-income country
NMFI: non-malarial febrile illness
NICE: National Institute for Clinical Excellence
PAM: potential available market
PPP: purchasing power parity
RDT: rapid diagnostic test
SAM: serviceable available market
SOM: serviceable and obtainable market
TAM: total available market
USD: United States dollar
